# Dignity in childbirth: A perspective from sub‐Saharan Africa

**DOI:** 10.1002/puh2.146

**Published:** 2023-12-21

**Authors:** Mirriam Window, Mama Tamanda Msiska, Symon Fidelis Nayupe, Gaily Lungu

**Affiliations:** ^1^ Department of Reproductive Health School of Maternal, Neonatal and Reproductive Health Kamuzu University of Health Sciences Blantyre Malawi; ^2^ Department of Nursing Nkhotakota District Hospital Nkhotakota Malawi; ^3^ Department of Medical Laboratory Sciences University Private Clinic, Kamuzu University of Health Sciences Blantyre Malawi; ^4^ Department of Midwifery School of Maternal Neonatal and Reproductive Health Kamuzu University of Health Sciences Blantyre Malawi

## INTRODUCTION

Childbirth is an important experience in a woman's life and a transition to motherhood. Dignity in childbirth entails that mothers be treated with utmost respect during the antenatal, childbirth and postnatal period to achieve such respect and the safety of both the mother and the child [1]. Proper and good quality maternity care improves maternal and neonatal outcomes.

Globally, attention has been given to observing and studying the treatment of women during childbirth as a significant contributor to inequalities and undignified treatment during access to healthcare [2]. Subsequently, there have been significant improvements in maternal health outcomes due to increased efforts to ensure that childbirth is handled with dignity [3]. This is manifested by a 45% decline in the global maternal mortality ratio from 380 to 210 per 100,000 live births [4]. Sub‐Saharan Africa (SSA) has also achieved a decline in maternal mortality rate (MMR) since 2000 with interventions such as encouraging healthcare deliveries and other safe delivery community health models [5, 6]. However, a comparative view of the MMR in SSA countries against high‐income countries still shows a high disparity, with SSA recording an MMR of 462 per 100,000 live births compared to 11 per 100,000 live births as of 2017 [7].

The causes of MMR are multiple, but among them is disrespectful and undignified care, which results in poor‐quality service or, in some cases, completely discourages women from attending maternity care altogether. Thus, respectful and dignified care in childbirth is paramount to ensure the mother's quality of service and safety and, second, as a right to maternal health [7, 8].

We discuss the dignity in childbirth in the context of SSA. We explore the impact of undignified care on childbirth and maternal health and provide recommendations on strengthening or improving dignified care in the region.

## THE CONCEPT OF DIGNITY IN CHILDBIRTH

Dignity and respect in childbirth go hand‐in‐hand with the ethical practice of respect for individuals in healthcare delivery. In pregnancy and childbirth, this means the respect women receive as they access perinatal health services. This entails that women and their partners be accorded the right to make decisions and control their maternal health, even when such decisions do not agree with the healthcare provider's point of view [1]. To the healthcare provider, this entails women being treated equally, deserving of privacy and given the necessary support in a manner that does not undermine women's autonomy.

## IMPACT OF LACK OF DIGNITY ON MATERNAL AND NEONATAL MORTALITY RATES

Lack of dignity can contribute to maternal and neonatal mortality in several ways. First, it compromises the quality of care. Healthcare practitioners who do not respect pregnant mothers are likely to neglect them, offer substandard services and impose decisions on expectant mothers against their will. Such behaviours put women at risk of birth complications such as haemorrhage and infections, which are some of the leading causes of maternal deaths in many SSA countries [9, 10, 11]. Furthermore, systemic inadequacies have also contributed to poor quality of care. A study in Migori, Kenya, revealed that mismanagement and poor‐quality care contribute to maternal and neonatal mortality on top of client failures [11]. Several other studies have also demonstrated how systemic quality challenges bordering on undignified care contribute to neonatal mortality in SSA [10, 11, 12].

Second, mothers’ experiences of undignified treatment may lead to them shunning seeking care at healthcare facilities and opting for traditional birth attendants, where they are at more risk of complications [12, 13].

## FACTORS CONTRIBUTING TO UNDIGNIFIED CARE

Undignified care would take the form of lack of privacy due to poor infrastructure, insufficient or improper care due to lack of resources or general poor treatment by healthcare workers due to bad attitudes or discriminatory treatment based on the socio‐economic status of expectant mothers.

### Attitude of healthcare workers

Healthcare workers’ attitudes play a crucial role in delivering services to mothers. A study done in SSA on the perception of women regarding respectful maternity care during facility‐based childbirth showed that three quarters of the women received respectful maternity care [13]. Although most women reported receiving respectful care, some reported verbal and physical abuse [13]. Sometimes, healthcare workers have negative attitudes towards some services women seek; an example is women seeking safe abortions who are sometimes faced with disrespect and undignified care from health workers. This is sometimes due to laws on abortion, which range from highly restrictive to more liberal in some parts of SSA [14, 15]. For instance, South Africa, Mozambique, Ethiopia and Ghana allow for abortion on request from 12 to 16 weeks of pregnancy; in countries like Malawi, Kenya, Tanzania and Uganda, abortion is only allowed when a woman's life or her health is at risk [14, 15]. As such, women tend to seek unsafe abortion procedures, and once they seek proper medical care due to complications that arise, they are stigmatised disrespected and end up receiving undignified care.

### Literacy and socio‐economic status of pregnant mothers

In communities with low literacy levels, decision‐making on childbirth choices on the part of mothers may be challenging. The temptation for healthcare workers to make directed decisions on these women due to their lack of understanding is common. The autonomy of non‐educated mothers is often overrun [16]. Additionally, some healthcare workers have ill attitudes towards women of low socio‐economic status of women. Women from low socio‐economic backgrounds who often deliver in public facilities have reported a lack of privacy and verbal abuse, unlike their counterparts who seek care in private facilities [13].

### Limited healthcare resources

Many public healthcare facilities in SSA continue to face the challenge of limited resources, a factor that hugely affects the delivery of services by healthcare professionals. Although nurses and midwives have good intentions and the goodwill to offer service to the best standards concerning dignity, such as privacy, this is barely possible in some institutions. With limited resources, hospitals may not invest in infrastructure designed to achieve privacy. With limited consumables, healthcare workers have to ask mothers to buy their necessities for childbirth, from gloves to maternity pads [17]. If a mother cannot afford this, it is a big challenge to achieve care during childbirth that would be of dignity to the mother and the child.

Where no private rooms are available, women are forced to stay in general wards where privacy is nearly non‐existent. Furthermore, privacy could be seen from a gender context concerning human resources in healthcare, when pregnant women may prefer female to male midwives. In general, except for institutional differences, a majority of countries in SSA have a predominantly female midwife workforce [18, 19, 20].

## RECOMMENDATIONS FOR DIGNIFIED CARE IN SSA

### Allocation of skilled personnel and patient management audit systems

To improve care, more skilled personnel are needed in maternal care units, especially in government institutions. In addition, maternity care facilities need an effective system to track whether patients are treated with respect, consideration and professionalism. To achieve this goal, staff attitudes towards patients and how patients are treated must be a significant consideration in hiring and retention, and abusers must be reported to regulatory bodies in the most severe cases [21]. There is also a need to establish and implement a reporting and response system to ensure that principles of respectful care and gender responsiveness are understood and applied [21].

### Community awareness and sensitisation

Communities should also be engaged in promoting dignity and respect in childbirth. This can involve creating awareness and sensitisation of the importance of dignity and respectful maternity care and informing women of their rights during childbirth. This can empower women to demand digital respect during childbirth.

### Fostering client feedback culture

Healthcare providers should develop a culture of requesting client feedback on their childbirth experiences. This can be done by conducting exit interviews in the maternity care facilities, placing suggestion boxes and conducting audits on the dignity of care. The feedback can improve dignity and respect in maternal health services, eventually improving maternal and neonatal health outcomes.

### Healthcare training

Healthcare providers should receive training and education on providing dignified and respectful care during childbirth (Table 1) [22]. This can include pre‐service, in‐service and on‐the‐job training on communication skills, empathy and cultural competencies when providing maternal health services.

**TABLE 1:** Factors driving non‐dignified care and recommendations to promote dignified midwifery care.

Factors driving non‐dignified care	Challenges	Recommendation
Healthcare worker attitudes	Poor attitudes of healthcare workers	Establishing reporting and response systems for dignified care Train healthcare workers on respectful maternity care Conducting care audits
Literacy and socio‐economic status	Low literacy level or socio‐economic status of women	Sensitisation and awareness campaigns on the rights of a woman in labour Advocate for legal reforms on abortion
Limited healthcare resources	Poor/no proper infrastructure in public facilities	Improving infrastructure

### Resource mobilisation and improvement in healthcare infrastructure

Countries in SSA should ensure that necessary equipment and materials for childbirth are available in all public healthcare facilities. This will reduce the financial burden on mothers and address the social and economic disparities when women seek dignified maternity care. Furthermore, there should be investments in healthcare infrastructure to provide private rooms and spaces that support privacy during childbirth, which would help women to have a more meaningful experience of maternity care.

### Advocating for legal reforms

Lastly, there is a need to advocate for legal reforms in abortion laws in countries with highly restrictive laws to provide safe and more dignified abortion services. Such laws will reduce the stigma associated with abortion, ensuring that women who seek abortion services receive respectful and dignified care.

## CONCLUSION

Respectful and dignified maternity care is critical in delivering maternity care in the parietal period. Dignified maternity care helps to promote constant and effective communication between the midwife and the clients/guardians of the clients. There has been significant progress in ensuring dignity in childbirth in SSA through providing health education to women, the empowerment of women on sexual and reproductive health, targeted healthcare interventions for access to good healthcare services for pregnant women and improvement of healthcare systems to ensure mothers get dignifying care. However, significant challenges such as poor attitudes by healthcare workers, poverty and illiteracy and limitations of resources, particularly in public healthcare institutions, block the attainment of absolute dignity in childbirth. Allocation of skilled personnel and patient management audit systems, community awareness and sensitisation, health worker training, infrastructural improvement and legal reforms on abortion are some areas to focus on now and in the future to achieve dignity for our mothers.

## AUTHOR CONTRIBUTIONS

Conceptualisation and designing the commentery; M.W,TMM,SN,G.L. Drafting the article and revising it critically: M.W,TMM,SN,GL; Supervising the article G.L. All authors approved the final version submitted.

## CONFLICT OF INTEREST STATEMENT

The authors declare no conflicts of interest to any party.

## FUNDING INFORMATION

This did not receive any funding from any institution or organisation.

## ETHICS STATEMENT

None.

## Data Availability

Data sharing not applicable to this article as no datasets were generated or analysed during the current study.
